# Coupled Bimanual Training Using a Non-Powered Device for Individuals with Severe Hemiparesis: A Pilot Study

**DOI:** 10.4172/2329-9096.1000404

**Published:** 2017-04-28

**Authors:** Preeti Raghavan, Viswanath Aluru, Sina Milani, Peter Thai, Daniel Geller, Seda Bilaloglu, Ying Lu, Donald J Weisz

**Affiliations:** 1 Department of Rehabilitation Medicine, New York University School of Medicine, New York, NY, USA; 2 Promotion of Research Involving Innovative Statistical Methodology (PRIISM), Steinhardt School of Culture, Education, and Human Development, New York, NY, USA; 3 Department of Neurosurgery, Mount Sinai School of Medicine, New York, NY, USA

**Keywords:** Flexor synergy, Stroke rehabilitation, Medical device, Arm function, Rehabilitation, Motor impairment, Fugl-Meyer scale

## Abstract

**Background:**

Few options exist for training arm movements in participants with severe post-stroke hemiparesis who have little active range of motion. The purpose of this study was to test the safety and feasibility of training with a non-powered device, the Bimanual Arm Trainer (BAT), to facilitate motor recovery in individuals with severe hemiparesis. The BAT enabled coupled bimanual training of shoulder external rotation, which is reduced in individuals with severe post-stroke hemiplegia. The rationale for bimanual training was to harness contralesional cortical activity to drive voluntary movement in the affected arm in patients who could barely perform unimanual movements.

**Methods:**

Nine participants with post-stroke hemiparesis, preserved passive range of motion and Modified Ashworth score of <3 in the shoulder and elbow joints, trained with the device for 45 minutes, twice a week for six weeks, and were assessed pre- and post-training.

**Results:**

All participants tolerated the training and no adverse events were reported. Participants showed significant improvement in the upper extremity Fugl-Meyer score post-training with an effect size of 0.89. Changes in the flexor synergy pattern accounted for 64.7% of the improvement. Improvement in active range of motion in the paretic limb occurred for both trained and untrained movements. Some participants showed improvement in the time taken to perform selected tasks on the Wolf Motor Function Test post-training.

**Conclusion:**

The results demonstrate the safety and feasibility of using the Bimanual Arm Trainer to facilitate motor recovery in individuals with severe hemiparesis.

## Introduction

Stroke is the leading cause of disability in the United States at a cost of $36.5 billion annually [1]. Approximately 48% of men and 60% of women who survive stroke have severe impairment [2]. This contributes greatly to the economic burden of stroke from lost productivity and increased health care costs. In addition, the incidence and prevalence of stroke in young adults between 45–64 years of age is increasing globally [3]. Over 65% of stroke survivors have persistent deficits in arm function beyond 6 months [4] which contributes greatly to disability, reduced quality of life, and increased health care costs. Therefore there is an urgent need to find solutions to effectively rehabilitate the arm after stroke.

Recent imaging studies show how recovery processes unfold after a stroke [5]. Early in recovery, the undamaged contralesional hemisphere shows increased activation [6–9], but eventually normal sensorimotor lateralization is restored in the stroke-affected hemisphere in patients who have recovered function in the affected arm [10–12]. Importantly, increases in neural activity in the contralesional motor areas in the first weeks after stroke correlate with better motor recovery in humans [13,14], and monkeys [9]. Persistent activation of the motor and non- motor areas in the contralesional hemisphere is however noted in patients with poor motor outcome [10,15]. A recent longitudinal case study of a patient’s recovery over 21 months revealed continuous change in activation in the contralesional hemisphere with concomitant improvement in motor performance, whereas the ipsilesional hemisphere demonstrated significant change only towards the end of the study period [16]. Furthermore, somatosensory and visual information from each side of the body is processed bilaterally [17–19], and interlimb coordination is mediated by motor representations in the parietal and premotor areas shared by both limbs [20]. In addition, disruption of activity in the dorsal premotor cortex of the intact hemisphere results in degraded behavior in the paretic hand [21]. Taken together, these studies suggest that actions from each arm are represented bilaterally, and redundant homologous pathways in the intact hemisphere can facilitate reorganization of the central nervous system to facilitate planning and control in the affected arm and hand post stroke.

How can the increased contralesional cortical activity be harnessed to drive voluntary movement in the affected arm in patients who have severe stroke and are unable to perform unimanual movements with their affected arm? At least two kinds of bimanual training protocols have been designed to harness contralesional cortical activity for post-stroke motor recovery. In active bimanual training, both arms move independently and simultaneously, requiring that individuals have some active movement on the paretic side. Active bimanual arm training combined with rhythmic auditory stimulation (BATRAC protocol, Tailwind device) led to increased recruitment in the contralesional and ipsilesional hemispheres with concomitant improvement in performance of the paretic arm [22,23]. In active-passive bimanual training, the non-paretic arm drives movements of the paretic arm and leads to simultaneous mirror movements of both arms. Here, bimanual training without auditory stimulation was used to prime the ipsilesional motor cortex for subsequent training with the paretic arm, and also led to significant gains in arm function [24–27]. An advantage of the active-passive approach is that it requires little active movement in the paretic arm; hence it can be used in individuals with significant paresis.

One question that arises is: which movements should be trained first after a stroke? Twitchell [28], Brunnstrom [29], and Fugl-Meyer [30] described a hierarchical progression of recovery from flaccid paralysis of the arm to return of reflex activity and the emergence of stereotypical flexor and extensor synergy patterns of movement along with muscle spasticity. Twitchell and others noted that the earliest movement that occurs in individuals recovering from hemiplegia is shoulder internal rotation. More recently investigators have quantified muscle synergies, which represent patterns of muscle activation with distinct spatial characteristics, in healthy individuals and patients with stroke, and found that muscle synergies involving proximal muscles exhibited consistent alterations following stroke. In particular patients with severe arm motor impairment show abnormally increased activation of the pectoralis major, which internally rotates and adducts the shoulder, and coactivation of the deltoid muscles [31]. Recruitment of the altered shoulder muscle synergies was strongly associated with abnormal task performance. Clinically, it is extremely difficult to alter the movement patterns of a patient who initiates voluntary movement by internally rotating the shoulder because it orients the upper arm, forearm, and hand towards the midline of the body, making functional movements, which require the arm and forearm to be oriented parallel to the body, extremely difficult. We surmised that a good place to begin would be to train individuals out of shoulder internal rotation. Shoulder external rotation is a difficult movement for severely paretic patients with stroke to perform by themselves. Lack of this movement prevents the forearm and hand from achieving a neutral position to perform other movements or activities of daily living. Therefore we designed the Bimanual Arm Trainer to primarily train shoulder external rotation.

The purpose of this study was to demonstrate the safety and feasibility of using the Bimanual Arm Trainer (BAT), a non-powered mechanical device, to provide coupled shoulder external rotation training whereby the non-paretic arm moves the paretic arm, to facilitate motor recovery in individuals with severe hemiparesis.

## Methods

### Participants

Nine participants (four females and five males, mean age ± SE = 55.5 ± 2.8 yrs) with severe post-stroke hemiparesis as determined by their baseline Fugl-Meyer scores (11.9 ± 2.8) participated in the study (Table 1). The participants were referred from the outpatient services at New York University Medical Center. Informed consent approved by the local institutional review board was obtained as per the Declaration of Helsinki (Clinical trial # NCT01422005). All participants had had varying amounts of rehabilitation services prior to participating in the study and had been discharged at the time of enrollment into the study. The inclusion criteria were: 1) ability to follow instructions in English; 2) ability to comply with the therapy protocol; and 3) likely to complete all study visits.

Participants were excluded if they had 1) severe upper extremity spasticity (Ashworth score of >3 at any joint), or evidence of joint contracture that precluded them for using the BAT; 2) evidence of alcohol, drug abuse or other relevant neuropsychiatric condition such as psychotic illness or severe depression; 3) history of surgery or other significant injury to either upper extremity causing mechanical limitations that preclude task performance; 4) previous neurological illness such as head trauma, prior stroke, epilepsy, or demyelinating disease; 5) complicating medical problems such as uncontrolled hypertension, diabetes with signs of polyneuropathy, severe renal, cardiac or pulmonary disease, or evidence of other concurrent neurologic or orthopaedic conditions precluding the subject from complying with the study protocol.

### Assessments

Feasibility to facilitate motor recovery was measured using standard neurological and musculoskeletal evaluation pre- and post-training with the device: upper extremity motor impairment was assessed using the upper extremity component of the Fugl-Meyer Scale (FMS) [30]; active range of motion (AROM) at the shoulder, elbow, forearm and wrist joints [32] were measured using 3D electromagnetic motion sensors sampled at 120Hz (The Motion Monitor, Innsport, Chicago); and the Wolf Motor Function Test was used to assess participants’ functional ability [33].

The Motion Monitor 3D electromagnetic motion sensor system used for measuring active range of motion utilizes the Ascension 800 sensor which has a static resolution of 0.5 mm for position and 0.1° for angular orientation, with an accuracy of 1.4 mm RMS for position and 0.5° RMS for angular orientation [34]. The participants were instructed to perform the following movements actively: shoulder internal and external rotation, shoulder abduction, shoulder flexion and extension, elbow flexion and extension, forearm pronation and supination, and wrist flexion and extension. The start position for each movement was fixed as follows: the shoulder and elbow movements shared the same start position with the subject sitting with their torso straight and arms down by the sides and elbows extended; for the forearm and wrist movements, the participants started with the elbows flexed to 90 degrees and the forearm in neutral (i.e. thumb facing the ceiling). The participants moved actively to the maximum possible range (peak) and returned to start position. The onset and offset of the movements were defined as the amplitude of the movement at 5% of peak angular velocity. All participants were not able to attain the desired start and end positions, and performance varied greatly depending on the start position of the joint. Therefore the range of motion for each joint from onset to peak and peak to offset was analyzed separately.

### Description of the Bimanual Arm Trainer (BAT)

**Figure 1:**
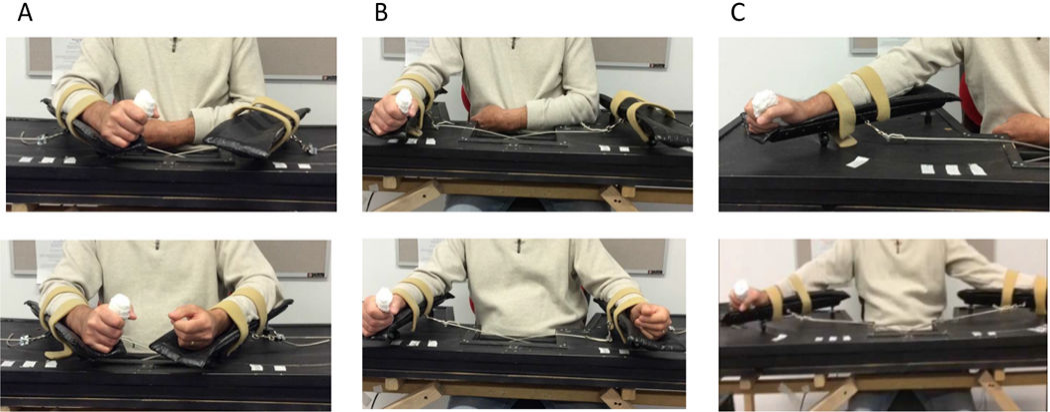
Bimanual Arm Trainer with affected arm only in device (top panel) and both arms in device (bottom panel) in (A) closed position (shoulder internally rotated and elbow flexed), (B) midway between open and closed position, and (C) open position (shoulder externally rotated, upper arm slightly abducted, and elbow partially extended).

The BAT facilitates training of shoulder external rotation in the paretic arm by moving the non-paretic arm. It is designed to restore balance between the muscles of the chest in the front and the muscles in the upper back. No active movement ability is required in the paretic arm to use the device. The paretic and non-paretic forearms are placed on connected movable troughs with the axis of rotation of the trough at the subject’s elbow (Figure 1). When the non-paretic arm moves the troughs outwards, both the non-paretic and paretic arms simultaneously externally rotate the shoulder with minimal torque and resistance. Note that the upper arm is slightly abducted (~30°) for comfort, and the elbow is partially extended in the open position as shown in Figure 1C. Since this is a non-powered device, the extent of movement of the affected arm is self-determined and is less likely to lead to injury.

### Training

Participants received 45 minutes of training using the BAT twice a week for 6 weeks. The height of the chair was adjusted such that the participants’ forearms were at the level of the elbow when positioned on the device. First the paretic arm was placed on the device and active movements were encouraged for 1–2 minutes. The maximum active range of motion was marked on the device.

Then the non-paretic arm was placed on the device which linked the two arms such that any movement of the non-paretic arm produced symmetric and simultaneous movement of the paretic arm. The participants were instructed to move their arms at a self-selected pace. The training began with the non-paretic arm doing 100% of the work and gradually progressed to increasing levels of work with the paretic arm. Rest breaks were given during training, and fatigue and comfort levels were monitored. No additional assistance was required during the training for 45 minutes. At the end of the session, participants first removed the non-paretic arm and performed active movements once again using just the paretic arm. This allowed participants to check their own progress from the beginning to the end of the session and provided motivation to return for training.

Safety of training with the BAT was assessed by enquiring about discomfort and fatigue in the affected and unaffected arms before and after training, and checking for adverse effects at each visit. We were particularly interested in signs of overuse injury, fatigue, and reduction of range of motion in the unaffected arm, and signs of skin breakdown in the affected arm from traction and friction with the device surface during training.

### Data analysis

Safety of using the BAT was analyzed qualitatively. Feasibility of the BAT in facilitating motor recovery was measured by change in the Fugl-Meyer scores (primary outcome measure). Secondary outcomes included active range of motion from onset to peak and peak to offset from pre-to post-training, and time taken on the Wolf Motor Function Test. The purpose of the secondary analyses was to quantify the change in motor patterns and function. Data analysis was performed using Rstudio (version 0.99). Due to the small sample size, and to avoid violation of normality assumption, the nonparametric Wilcoxon Signed Rank Test tests were used for inference. We report the effect size (pre/post change divided by SD) of the tests as these are valid to test outcomes irrespective of the sample size [35]. One subject (1251) could not perform the post-training assessments due to injury to the affected hand unrelated to the study and wore a cast on the affected arm for the post-training assessments.

Some participants were unable to perform certain movements or performed the opposite movement during active range of motion assessments (19/99 movements); the data from these movements pre-and post-training were excluded from analyses. Percent symmetry was calculated by the ratio of the range of motion on the affected side/ unaffected side for each subject and expressed in percentage.

## Results

**Figure 2:**
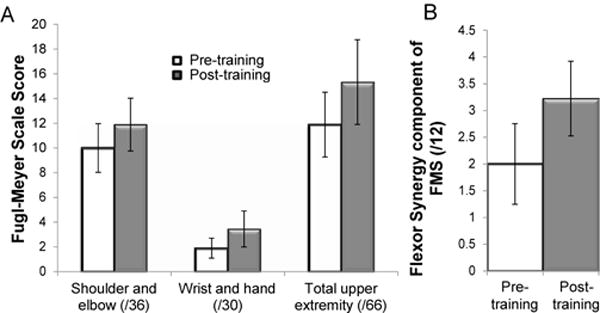
(A) Mean (± SE) Fugl-Meyer scores from pre- to post-training, (B) Mean (± SE) Change in the flexor synergy score on the Fugl-Meyer scale from pre- to post-training.

All participants tolerated the training. No adverse events were reported. The primary outcome measure was change in upper extremity Fugl-Meyer scores from pre- to post-training. There was a significant improvement in the mean pre-post difference (± SE) of the total upper extremity Fugl-Meyer score of 3.4 ± 1.4 points after 6 weeks of training (Wilcox Signed Rank Test Statistic W=35, p=0.043, Cohen’s effect size d=0.89). Figure 2 shows the changes in the total upper extremity Fugl-Meyer score, as well as in the shoulder/elbow and wrist/hand scores (Figure 2A). We found that the change in the flexor synergy component of the shoulder/elbow score accounted for 64.7% of the pre-post difference (Figure 2B, W=42, p=0.019, d=0.93). There was no significant correlation between the time since stroke and change in Fugl-Meyer scores.

Active range of motion was measured in both the non-paretic and paretic upper limb joints. Since participants could not all attain the desired start and end positions, the peak angle, as well as the range of motion from onset to peak and peak to offset (or return to start position) were examined. Since the peak angle varied greatly depending on the start position, statistical analyses were only performed for the range of motion from onset to peak movement and peak to offset (Table 2). As expected, the range of motion in the non-paretic upper limb was greater than in the paretic upper limb. There were no substantial differences in the range of motion of the non-paretic upper limb after training on the bimanual arm trainer. On the paretic side, on average, the range of shoulder internal rotation from onset to peak was unchanged but return to start position by shoulder external rotation (peak to offset) was improved post-training (d=0.81), suggesting that the upper arm could be held in a more neutral position, rather than in an internally-rotated position (Table 2). Return to start position from peak external rotation was also improved (d=0.78), suggesting greater control in both directions at the joint. Note that the percent symmetry for shoulder external and internal rotation increased substantially post-training, suggesting that the movements were more similar to those on the non-paretic side after training on the bimanual arm trainer.

Untrained joints also showed changes from pre-to-post training even though there was greater between subject variability as noted in the standard error (Table 2). Overall, there was an increase in elbow extension on return from peak elbow flexion of 9.1 ± 14.6 degrees from pre- to post-training, but when participants were asked to extend their elbow from the start position there was a 15.9 ± 14.5 degree reduction in extension. A closer examination of the data reveals that after training, some participants (1357,1398) tended to hold the elbow in a substantially more extended than flexed position at rest, reducing the amount of excursion on extension.

Interestingly, the range of active forearm pronation improved both from start to peak pronation (d=1) and from peak supination back to start position (d=0.86). The range of wrist extension from peak wrist flexion to start position also improved (d=0.71).

Participants showed improvement in either the peak angle, or the range of motion at several joints. In some cases the improvement was in both directions, whereas in others it was preferentially in one direction. The paretic upper limb joints that showed improvement for each participant are listed in Table 1. All participants with available data for the joint showed increased shoulder external rotation as a direct effect of training.

**Figure 3:**
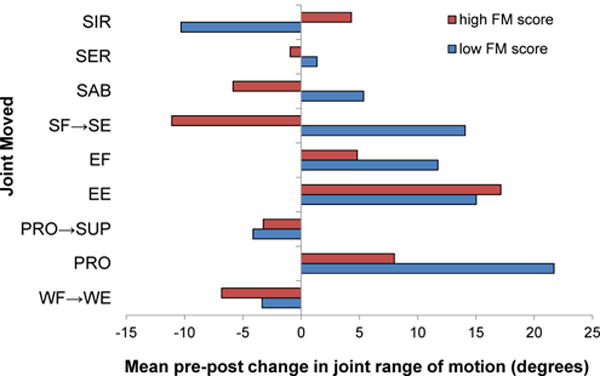
The bars represent the mean change in joint range of motion from pre-training to post-training in participants with low Fugl-Meyer (FM) scores (n=4) vs. those with high FM scores (n=5). The blue bars represent the change in participants with low FM scores, whereas the red bars show the change in participants with high FM scores. SIR=shoulder internal rotation, SER=shoulder external rotation, SAB=shoulder abduction, SF→SE=shoulder flexion to extension (retraction), EF=elbow flexion, EE=elbow extension, PRO→SUP=pronation to supination, PRO=pronation, WF→WE=wrist flexion to extension.

Qualitatively, participants with low Fugl- Meyer scores (first 4 in Table 1), showed greater decrease in shoulder internal rotation, increase in shoulder external rotation, shoulder abduction, shoulder extension (retraction), elbow flexion, forearm supination (from a pronated position) and pronation (Figure 3, blue bars).

In contrast, participants with higher Fugl-Meyer scores (last 5 in Table 1), showed greater increase in shoulder internal rotation, elbow extension, and wrist extension (from a flexed position) (Figure 3, red bars).

**Figure 4:**
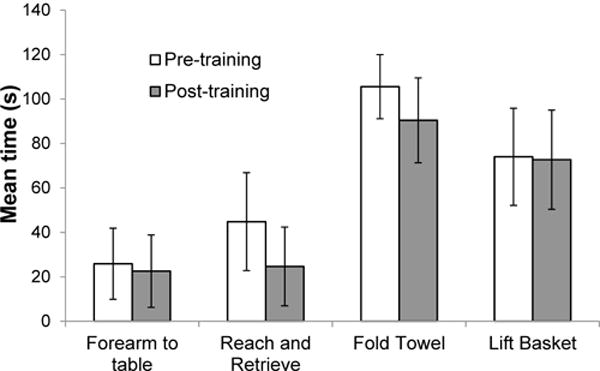
Mean time taken to perform four tasks on the Wolf Motor Function Test ± SE (n=7).

We then asked if the changes in active movement had functional consequences by examining performance on the Wolf Motor Function Test. Most participants were able to perform only 10 tasks in the test battery (forearm to table, forearm to box, elbow extension, elbow extension with weight, hand on table, hand on box, hand on box with weight, reach and retrieve, fold towel, and lift basket), consistent with the severity of their motor impairment. Participants had difficulty with tasks that required more hand and finger movements (lift can, lift pencil, lift clip, stack checkers, flip cards, and turn key). The tasks that showed improvement for each subject are noted in Table 1. More participants showed improvement across four tasks (Figure 4), but particularly for reach and retrieve and fold towel, with a significant reduction in average time taken from 62.57±17.42 s pre-training to 52.60±17.12 s post-training.

## Discussion

The purpose of this study was to demonstrate the safety and feasibility of using the Bimanual Arm Trainer (BAT), a non-powered mechanical device to provide coupled shoulder external rotation training whereby the non-paretic arm moves the paretic arm, to facilitate motor recovery in individuals with severe hemiparesis. We found that the participants tolerated training without adverse effects to the non-paretic and paretic arms. The relatively small dose of training produced a clinically important change in motor impairment on the Fugl-Meyer scores as well as increased active range of motion at some trained and untrained joints.

### Usefulness of bimanual training

Bimanual training has been found to be efficacious in reducing proximal upper limb impairment and improving motor kinematics particularly in patients with moderate to severe hemiparesis [36,37]. Therefore the changes in Fugl-Meyer scores and active range of motion noted in this study are concordant with published results. Bimanual movements that require simultaneous homologous movements have been shown to decrease cortical inhibition and enhance cortical motor activity in both hemispheres, with increased plasticity for trained movements [38,39].

Bimanual training can be applied to one joint e.g. to the wrist [40], or to movement at more than one joint performed together, e.g. shoulder flexion and elbow extension as with the BATRAC protocol [22]. It is conceivable that each of the devices and protocols could be useful in particular patients given individual patterns of impairment and the specific rehabilitation goals. The BAT device used in this study was designed primarily to train shoulder external rotation in participants with severe hemiparesis, who often become progressively internally rotated over time. However the training was by no means entirely restricted to this joint as the participants also extended their elbow during training. Our results show that after training, the participants improved their ability to externally rotate the shoulder to a neutral position from an internally rotated position. In addition several untrained movements also improved after training with the BAT. The results suggest that the training can generalize to more distal joints.

### Changing abnormal patterns of movement

The logic behind training with the BAT was that since abnormally increased internal rotation is a hallmark of the movement pattern in individuals with hemiparesis [31], training external rotation may facilitate movement out of this pattern. Brunnstrom described stereotypical stages of motor recovery from flaccid paralysis, to the development of spasticity with synergistic patterns of movement, and finally to voluntary motor control that is not limited by synergistic patterns of movement [41]. Subsequently, Fugl-Meyer followed a cohort of hemiplegic patients from one week post-stroke throughout one year, and developed the Fugl-Meyer scale to document a definable course of motor recovery through this sequential pattern from synergistic to isolated movements [30]. It was postulated that patients could progress from one recovery stage to the next at variable rates, but always in an orderly fashion without omitting any stage, although recovery may be arrested at any stage. The Fugl-Meyer scale has been shown to have excellent reliability [42], and is still the most widely-used measure of motor recovery post stroke [43,44]. However it is still not known what mediates the progression of motor recovery from one stage to the next, and what can be done to facilitate such progression. Conceivably the improvement in movement pattern with training would depend on the stage of recovery of the individual at the time of training.

The first part of the Fugl-Meyer scale examines the flexor synergy, which requires shoulder elevation, retraction, abduction, external rotation, elbow flexion and forearm supination, and the second part examines the extensor synergy which requires shoulder adduction, elbow extension, and forearm pronation. Our results show that patients with a low Fugl-Meyer score at the beginning of training showed greater improvement in shoulder external rotation, shoulder abduction, shoulder retraction, elbow flexion, and forearm supination and pronation, consistent with a progression from the flexor synergy to the extensor synergy. Patients with higher Fugl-Meyer scores showed greater increase in shoulder internal rotation, elbow extension, and wrist extension which are required to perform tasks that combine the flexor and extensor synergies and enable movements out of the synergy pattern such as bringing the hand to the spine, as well as more distal movements. The pattern of improvement in joint motions seen in this study suggests that coupled training of shoulder external rotation of both arms can lead to increased active movement across trained and untrained joints in patients with severe chronic hemiparesis, reflecting progression across the stages of motor recovery. The underlying mechanisms of such recovery may be more efficient harnessing of bilateral cortical [45] and spinal [46] connectivity.

### Function follows movement

Rehabilitation goals typically focus on function. However there can be no function (other than as a static holder) without movement. For patients with severe hemiparesis who have little active voluntary movement, restoration of active movement is the first goal followed by utilization of the available movements to perform functional activities. If patients with severe hemiparesis who do not have normal movements, are forced to move repeatedly without guidance on how to move, they will naturally reinforce their abnormal patterns of movement. During training with the BAT, however, the fixed movement track prevents the learning of abnormal compensatory strategies. External rotation at the shoulder is needed to maintain a neutral position of the upper arm so that the forearm is parallel to the midline of the body. This position is necessary to perform most functional tasks. All the participants showed reduced internal rotation and increased active external rotation as a result of training. Furthermore, most participants were able to keep their elbow more extended at rest, actively pronate their forearm, and also extend the wrist into neutral from a flexed position. These changes, as well as more subtle changes in movement for each subject may have contributed to improved performance on the Wolf Motor Function Test. These changes, though small, are meaningful to patients because it enables them to do things they couldn’t accomplish before. Thus training with the BAT may facilitate subsequent functional task training with the paretic hand to further improve arm and hand function.

### Facilitating movement across the continuum of recovery

Post-stroke recovery has been found to be most rapid in the acute (0–1 month) and subacute (1–6 months) phases [47], but it can continue well into the chronic phase (>6 months) [48]. The participants in this study were mostly in the chronic phase of recovery (only one subject was approaching 6 months post-stroke). However there was no correlation between change in the Fugl-Meyer score and time since stroke, suggesting that the BAT can potentially benefit patients even long after the stroke. One of the greatest barriers to motor improvement at any phase of recovery is the availability of therapy or training; only 30% of individuals who need rehabilitation actually get it [49], and there is increasing disparity in the availability of rehabilitation services 1 year post stroke [50]. Given that the prevalence of stroke is projected to increase significantly in younger individuals in the next two decades [1], with significant long-term disability, there is a dire need to facilitate long-term training.

The BAT can safely facilitate high-intensity training of movements that are critical for function, without the need for skilled supervision or external power. The utility of the BAT lies in its ability to facilitate repeated movements of the paretic shoulder and upper arm in a direction that is not easily trainable, providing the opportunity to gain a sense of control over one’s own rehabilitation. It is thus ideally suited to supplement traditional therapy in rehabilitation facilities, in community centers, and for home use. It may be used in individuals with unilateral paresis from stroke or any other form of brain injury, e.g. TBI or multiple sclerosis, or peripheral injury, e.g. brachial plexus injury that produces weakness on one side. One feedback that we received from participants is that interfacing the BAT with games on a computer may further motivate training with this device.

### Limitations

This is a single group pre/post study design without a control group and without randomization. The purpose of the study was to test the safety and feasibility of training with the device towards larger sample studies. Nevertheless the results are noteworthy as the study was performed in a cohort of severely impaired participants who were in the chronic stage post-stroke, and received only 12 sessions of training over a period of 6 weeks. Despite this small dose, the effect size for change in motor impairment, and motor pattern at specific trained and untrained joints was high to very high (>0.7), suggesting a strong effect of training.

## Conclusion

This study demonstrates the safety and feasibility of using the Bimanual Arm Trainer (BAT), a non-powered mechanical device, to provide coupled shoulder external rotation training whereby the non-paretic arm moves the paretic arm, to facilitate motor recovery in individuals with severe hemiparesis even in the chronic phase of recovery. Randomized controlled studies testing the effect of the BAT over a longer term are warranted.

## Figures and Tables

**Table 1:** Participant characteristics.

Participant	Age	Sex	Hemiparesis	Handedness	Time since stroke (months)	Baseline FM score (/66)	Joint movement improvements	Improved time on WMFT tasks
1246	59	Male	Right	Right	202.9	4	peak SER, SAB range, peak EF and EF range, EE range, EE→EF range, peak PRO and PRO range, PRO→SUP range, peak WF, WF→WE range	forearm to table
1296	47	Male	Left	Right	43.3	4	peak SER, SER range, SAB range, SAB→SAD range, peak SE and SE→SF range, peak EF, EF range, peak EE, EE→EF range, PRO range, PRO→SUP range, WF→WE range, peak WE, WE→WF range	forearm to table
1394	47	Female	Left	Right	17.9	4	peak SAB, SAB range, SE→SF range, EF→EE range, WE→WF range. Other joints not available due to poor movement quality	hand on table and reach and retrieve*
1400	67	Male	Right	Right	37.6	4	SIR→SER range, peak SER, SAB→SADD range, SF→SE range, peak SE, SE range, peak EF, EF range, EF→EE range, peak PRO, PRO range, SUP range, SUP→PRO range, peak WF, WF range, WF→WE range	forearm to table* and hand on table*
1357	57	Male	Left	Right	15.3	14	peak SIR, SIR range, SIR→SER range, peak SAB, peak SF, SF range, SF→SE range, peak SE, SE range, SE→SF range, PRO range, PRO→SUP range, peak SUP, SUP→PRO range, WF range, WF→WE range, peak WE, WE range, WE→WF range	forearm to table, hand on table, hand on box with weight, reach and retrieve, turn key*, and lift basket
1398	43	Female	Right	Right	21.4	15	peak SIR, SIR→SER range, SER range, SAB range, peak EE, peak PRO, PRO range, PRO→SUP range, SUP→PRO range, WF range, WF→WE range	not available
1251	51	Female	Left	Right	38.1	16	peak SF and SF range, peak SE and SE range. Other joints not available due to cast.	not available
1327	58	Male	Left	Left	5.7	19	SIR range, SIR→SER range, peak SER, SER→EIR range, SAB→SAD range, SF→SE range, peak SE, EF range, EF→EE range, peak PRO, PRO range, SUP→PRO range, peak WF, WF range, WF→WE range, peak WE, WE range	elbow extension, elbow extension with weight and lift basket
1338	66	Female	Right	Right	27.8	27	peak SIR, IR range, IR→ER range, SAB→SAD range, peak SF, SF range, SF→SE range, SE range, SE→SF range, EF range, EF→EE range, EE→EF range, peak PRO, PRO range, SUP range, SUP→PRO range, Peak WF, WF range	forearm to box, elbow extension with weight, hand on table, hand on box, reach and retrieve, fold towel* and lift basket

FM=Fugl-Meyer, SIR=shoulder internal rotation, SER=shoulder external rotation, SAB=shoulder abduction, SAD=shoulder adduction, SF=shoulder flexion, SE=shoulder extension, EF=elbow flexion, EE=elbow extension, PRO=pronation, SUP=supination, WF=wrist flexion. WMFT=Wolf Motor Function Test.

* Participant could not perform task pre-training.

**Table 2:** Active Range of Motion.

Joint motion	Phase of movement	Unaffected		Affected		Percent Symmetry	
		mean ROM (SE)		mean ROM (SE)			
		Pre-training	Post-training	Pre-training	Post-training	Pre-training	Post-training
Shoulder int. rotation	Onset to peak	39(4.8)	44.6(2.9)	25.5(5.2)	24.9(8.2)	53%	55%
(SIR→SER)	Peak to offset*	37.7(4.0)	42.7(5.4)	28(8.4)	34.0(9.6)	69%	84%
Shoulder ext. rotation	Onset to peak	33.5(4.6)	28.1(4.4)	19.9(3.9)	19.9(3.59	63%	77%
(SER→SIR)	Peak to offset*	35.2(5.5)	27.6(6.6)	22.6(7.3)	15.2(5.1)	57%	117%
Shoulder abduction	Onset to peak	124.6(11.0)	128.9(7.9)	35.9(7.1)	35.1(6.0)	30%	27%
(SAB→SAD)	Peak to offset	121.2(14.4)	129.7(7.9)	28.3(6.6)	32.3(6.5)	32%	25%
Shoulder flexion	Onset to peak	142.1(4.6)	143.9(4.9)	51.4(10.1)	46.1(12.1)	35%	32%
(SF→SE)	Peak to offset	150.2(5.7)	153.2(4.8)	45.6(9.1)	45.5(12.6)	29%	30%
Shoulder extension	Onset to peak	62.6(3.3)	67.1(5.3)	14.2(2.5)	13.5(3.5)	23%	23%
(SE→SF)	Peak to offset	76.5(5.0)	78.4(4.1)	16.4(3.6)	13.9(3.6)	21%	19%
Elbow flexion	Onset to peak	113.9(2.6)	118.1(5.6)	79.1(8.5)	87.3(6.0)	70%	74%
(EF→EE)	Peak to offset	117.7(4)	123.7(7.2)	50.6(17.2)	59.8(12.1)	42%	47%
Elbow extension	Onset to peak*	120.7(3.9)	125.6(5.6)	69.3(16.8)	53.4(14.9)	57%	42%
(EE→EF)	Peak to offset	122.9(3.3)	128.3(4.3)	39.2(20.1)	43.5(17.4)	31%	32%
Forearm pronation	Onset to peak*	86.6(4.1)	83.6(5.2)	24.8(6.9)	38.7(6.9)	28%	50%
(PRO→SUP)	Peak to offset	106.2(9.4)	99.7(6.9)	23(11.8)	26.6(10.5)	19%	27%
Forearm supination	Onset to peak	70.6(8.3)	67.7(8.3)	28.1(7.8)	29.6(7.1)	40%	45%
(SUP→PRO)	Peak to offset*	96.7(10.9)	81.2(9.9)	23.6(9.8)	34.5(10.1)	27%	39%
Wrist flexion	Onset to peak	68.9(8.3)	68.9(5.5)	18.9(7.5)	22.5(6.8)	26%	34%
(WF→WE)	Peak to offset*	76.6(10.2)	71.9(6.1)	21.2(9.1)	26.5(8.4)	31%	39%
Wrist extension	Onset to peak	44.1(4.0)	46.8(5.6)	19.1(6.7)	19.6(5.1)	37%	52%
(WE→WF)	Peak to offset	56.5(7.7)	59.3(4.9)	19.7(11.3)	14.0(6.2)	30%	23%

SIR=shoulder internal rotation, SER=shoulder external rotation, SAB=shoulder abduction, SAD=shoulder adduction, SF=shoulder flexion, SE=shoulder extension, EF=elbow flexion, EE=elbow extension, PRO=pronation, SUP=supination, WF=wrist flexion.

* Effect size of mean pre-post difference >0.7.
